# Cerebrospinal fluid pressure dynamics as a biomechanical marker for quantification of spinal cord compression: Conceptual framework and systematic review of clinical trials

**DOI:** 10.1016/j.bas.2025.104211

**Published:** 2025-02-12

**Authors:** Najmeh Kheram, Madeleine A. Bessen, Claire F. Jones, Benjamin M. Davies, Mark Kotter, Mazda Farshad, Markus Hupp, Daniel Nanz, Patrick Freund, Martin Schubert, Vartan Kurtcuoglu, Armin Curt, Carl M. Zipser

**Affiliations:** a Spinal Cord Injury Center and Department of Neurology and Neurophysiology, Balgrist University Hospital, University of Zurich, Zurich, Switzerland; b University Spine Center, Balgrist University Hospital, University of Zurich, Zurich, Switzerland; c The Interface Group, Institute of Physiology, University of Zurich, Zurich, Switzerland; d Adelaide Medical School, The University of Adelaide, Adelaide, Australia; e School of Electrical & Mechanical Engineering, The University of Adelaide, Adelaide, Australia; f Department of Neurosurgery, University of Cambridge, Cambridge, United Kingdom; g Department of Spine Surgery, Balgrist University Hospital, University of Zurich, Zurich, Switzerland; h Swiss Center for Musculoskeletal Imaging, Balgrist Campus AG, Zurich, Switzerland; i Medical Faculty, University of Zurich, Zurich, Switzerland; j Zurich Center for Integrative Human Physiology, University of Zurich, Zurich, Switzerland

**Keywords:** Cerebrospinal fluid pressure dynamics, Spinal cord compression, Spinal cord injury, Degenerative cervical myelopathy

## Abstract

**Introduction:**

In patients with acute spinal cord injury (SCI) and degenerative cervical myelopathy (DCM), spinal cord compression is considered a main contributor to spinal cord damage, associated with cerebrospinal fluid (CSF) space obstruction. CSF pressure (CSFP) dynamics are studied as a potential indirect biomechanical marker for spinal cord compression, and as a proxy to estimate spinal cord perfusion pressure (SCPP).

**Research question:**

Evidence for safety and feasibility of CSFP dynamics in clinical trials as well as interrelations with neuroimaging and intraspinal pressure, and relation to preclinical CSFP models.

**Material and methods:**

Systematic review. This review followed PRISMA guidelines, risk of bias assessment with ROBINS-I tool, PROSPERO registration (CRD42024545629).

**Results:**

11 relevant papers were identified (n = 212 patients, n = 194 intraoperative, n = 18 bedside). Risk of bias for safety reporting was low-moderate. Intraoperative CSFP assessments were commonly performed in acute SCI. CSFP was assessed to calculate SCPP (7/11), to evaluate effects from surgical decompression (5/11) and for therapeutic CSF drainage (3/11). The adverse event rate associated with the intrathecal catheter was 8% (n = 15/194).

**Discussion and conclusion:**

The preliminary safety and feasibility profile of CSFP assessments in spinal cord compression encourages clinical application. However, a deeper risk-benefit analysis is limited as the clinical value is not yet determined, given challenges of defining disease specific critical CSFP and SCPP thresholds. The interrelation between measures of CSFP and neuroimaging is yet to be proven. Targeted preclinical studies are essential to improve our understanding of complex CSFP-cord compression interrelations.

## Highlights

• Monitoring of CSF pressure dynamics is an emerging topic in spinal cord compression.
• Systematic review of safety and feasibility in spinal cord compression.
• Safety profile of CSF pressure monitoring encourages clinical utility investigation.
• Yet to provide a risk-benefit consideration as the clinical utility is undetermined.
• Major focus on spinal perfusion in SCI±CSF drainage and decompression.

**Table:** Abbreviations

**AE**	Adverse event
**AIS**	American Spinal Cord Injury Association Impairment Scale
**ASIA**	American Spinal Cord Injury Association
**CNS**	Central nervous system
**CSF**	Cerebrospinal fluid
**CSFP**	Cerebrospinal fluid pressure
**CSFPp**	Cardiac-related CSFP pulsations
**CT**	Computed tomography
**DCM**	Degenerative cervical myelopathy
**ECG**	Electrocardiogram
**ICP**	Intracranial pressure
**ISP**	Intraspinal pressure
**JOA**	Japanese Orthopedics Association
**MAP**	Mean arterial pressure
**MRI**	Magnetic resonance imaging
**PC-MRI**	Phase contrast magnetic resonance imaging
**pCO**_**2**_	Blood CO_2_ levels
**PRISMA**	Preferred Reporting Items for Systematic reviews and Meta-Analyses
**PROSPERO**	International prospective register of systematic reviews
**ROBINS-I**	Risk of bias in non-randomised studies – of interventions
**SAE**	Severe adverse events
**SCI**	Spinal cord injury
**SCPP**	Spinal cord perfusion pressure

## 1 Introduction

Cerebrospinal fluid pressure (CSFP) was initially considered a diagnostic procedure for assessing spinal cord compression before being abandoned in favor of non-invasive spine imaging (Curt et al., 2025). Challenges in diagnosis and treatment of degenerative cervical myelopathy (DCM) and acute spinal cord injury (SCI) led to a renewed clinical interest in CSFP dynamics in the late 2000s.

The rationale behind monitoring of CSFP in spinal cord compression rises from the close link between the central nervous system (CNS) and the cerebrospinal fluid (CSF) system, which contributes to the maintenance of the CNS homeostasis (Spector et al., 2015). CSF is in constant motion, featuring complex dynamics (Gupta et al., 2010). Superimposed onto its steady flow from the production (MacAulay et al., 2022) to absorption (Proulx, 2021) sites are oscillations caused by cardiovascular and respiratory action (Liu et al., 2024; Schmid Daners et al., 2012), as well as transient flow caused by body movement (Magnaes, 1976). Ependymal cilia further contribute to CSF dynamics (D'gama et al., 2024), though their effect appears to be small in human adults (Siyahhan et al., 2014). The pulsatile motion of CSF indicates the presence of driving pressure gradients in the CSF and, hence, differences in the instantaneous pressure of CSF at different locations (Linninger et al., 2007).

**Fig. 1:**
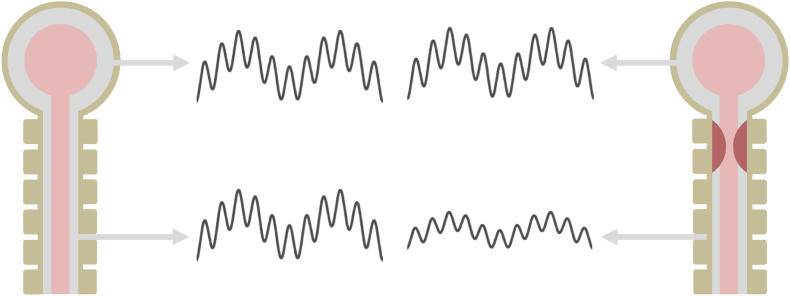
Conceptual illustration of the effect of spinal cord compression on the propagation of cardiorespiratory cerebrospinal fluid (CSF) pressure oscillations. Drawings are not to scale. Left: Normal cranial and spinal CSF compartments (grey: CSF; pink: brain and spinal cord; khaki: skull and vertebrae). The top and bottom curves represent, respectively, the time course of intracranial pressure (ICP) and CSF pressure (CSFP) characterized by cardiovascular and respiratory modulation. Phase shift between the two signals is omitted for simplicity. Right: Spinal compartment with intrathecal obstruction (red filled semicircles). For the same ICP time course, CSFP has reduced oscillation amplitude compared to the obstruction-free situation.

The intra- and extra-cranial CSF spaces are distinct in their properties but communicate under normal physiologic conditions. When cerebral blood volume increases in systole, CSF is expelled into the spinal subarachnoid space, freeing room within the cranial vault and limiting the elevation of intracranial pressure (ICP). In other words, this communication contributes to intracranial compliance (Avezaat et al., 1986a) (which is the inverse of the complementary metric, elastance (Avezaat et al., 1986b)). Similarly, cerebral blood volume decreases during inspiration due to increased venous outflow and thus decreases ICP (Yamada et al., 1975), causing CSF backflow to the cranium from the spinal compartment. Consequently, ICP oscillates with cardiac and respiratory cycles. These oscillations propagate to the spinal space and can be recorded as cardiac-related CSFP pulsations (CSFPp) and respiratory modulation. A lack of correspondence between ICP and CSFP waveforms may be indicative of altered communication between the cranial and spinal compartments due to intrathecal obstruction (Fig. 1) (Behrens et al., 2013). However, ICP and CSFP are rarely acquired simultaneously in the clinical setting. As an alternative, maneuvers that selectively perturb ICP may be used in combination with lumbar CSFP measurement: absence of, or substantially weaker, perturbation of CSFP would again be considered a reflection of obstruction. In patients with severe spinal cord compression, there is limited or no increase of CSFP downstream of the obstructed CSF space.

The primary aim of this systematic review is to evaluate the reported clinical trials for feasibility and safety of intraoperative or bedside CSFP dynamics monitoring in DCM and SCI. Its secondary aim is to contextualize CSFP dynamics with regards to preclinical evidence and alongside other methods for quantifying spinal cord compression, including MRI, ultrasound, and subdural (intraspinal) pressure (ISP) measurement. Although this review examines CSFP dynamics in both SCI and DCM due to their shared underlying mechanism of spinal cord compression contributing to neural damage, it is important to distinguish between these conditions, as they differ significantly in clinical presentation, pathophysiology, and treatment.

## 2 Methods

For the systematic review of clinical trials involving CSFP assessments, a literature search was conducted on Embase and MEDLINE using the terms [(cerebrospinal fluid pressure OR intrathecal pressure) AND (spinal cord injury OR degenerative cervical myelopathy)] on July 16th, 2024. Reporting followed the PRISMA guidelines (Page et al., 2021) where PICO model (standing for population, intervention, comparator and outcome) was used to construct the research questions: Population: adult patients with SCI or DCM; Intervention: cerebrospinal fluid pressure assessments; Comparator: neuroimaging and ISP; and, Outcome: feasibility and safety of CSFP assessments. Accordingly, the primary aim of the systematic review was to evaluate the evidence for safety and feasibility. Secondary aims were identifying the rationale for CSFP assessments in SCI and DCM, and the role of neuroimaging. Both interventional and observational studies were included, and case reports were excluded. All studies were rated for risk of bias by two raters (NK, CZ) with the ROBINS-I tool (Sterne et al., 2016), accounting for inclusion of both randomized and non-randomized cohort studies. In cases of disagreement, findings were discussed with team members and resolved by consensus. This review was registered on PROSPERO (CRD42024545629).

## 3 Results

### 3.1 Clinical trials of CSFP in SCI and DCM: study population and intervention

**Fig. 2:**
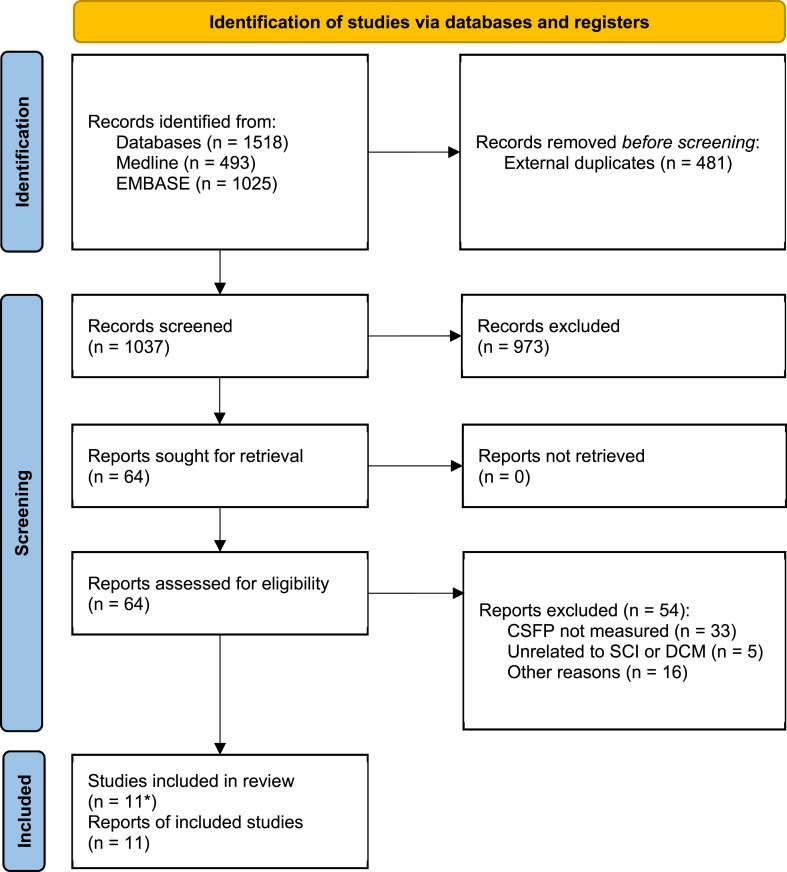
PRISMA flowchart for study inclusion. ∗10 studies were published within the last ten years, one seminal study was published before (Kwon et al., 2009). Other reasons for excluding reports were ineligible article types: comments, letters, case reports or reviews.

Eleven relevant papers (SCI: n = 186/212, 88%; DCM: n = 26/212, 12%) were identified for the questions of the systematic review (Kheram et al., 2022, 2023; Zipser et al., 2022; Theodore et al., 2023; Kwon et al., 2009; Altaf et al., 2017; Squair et al., 2017, 2019; Hogg et al., 2020; Yue et al., 2020; Kumar et al., 2023) (Fig. 2), with a total of n = 194 patients enrolled for intraoperative CSFP, and n = 18 patients enrolled for bedside assessments. There were seven single-center and two multicenter cohorts; three publications resulted from one multicenter cohort. Table 1 gives an overview of the included studies, their characteristics, and key findings. Most studies were conducted in the intraoperative setting (9/11 papers) and fewer at bedside (2/11 papers). Most intraoperative studies measured CSFP in SCI (8/11 papers, n = 175/194, 90%), while one intraoperative study enrolled patients with DCM (1/11 papers, n = 19/194, 10%). Most studies applied observational designs (7/11); the remaining used interventional designs (4/11): three investigated CSF drainage, and one explored drug effects. The intraoperative trials in SCI widely limited inclusion to American Spinal Cord Injury Association (ASIA) Impairment Scale (AIS) A-C, with a majority of AIS A-B patients included (i.e., patients with more severe neurological impairment). The surgical approach was reported in n = 98 perioperatively monitored patients, with mostly posterior approach (n = 74/98, 76%), followed by combined and anterior approach. For intraoperative CSFP assessments, all studies inserted intrathecal catheters at lumbar levels (commonly in anaesthetized patients, 4/9, only one study reported bedside insertion in awake patients; not reported in the other four studies) and continuously monitored for 1–5 days. For bedside testing, lumbar puncture was performed where the needle was attached to the monitoring device. CSFP recordings used various models of fluid-filled catheters (Braun Medical, Inc., US; Neuromedex®, Germany), drainage systems with integrated pressure transducers (Integra™, US; Medtronic GmbH, Switzerland; Neuromedex®, Germany), which were connected to various monitoring devices (Philips, Netherlands; SpaceLabs Healthcare, US; General Electric, US). All intraoperative studies also recorded mean arterial pressure (MAP), while bedside testing recorded electrocardiogram (ECG) in parallel.

**Table 1:** Study overview. ∗ same sample, different publications from Squair et al., 2017); ∗∗ same findings. Abbreviations: AIS: ASIA Impairment Scale; C-T-L: cervical- thoracic- and/or lumbar level of injury; CSFD: Cerebrospinal fluid drainage; CSFP: Cerebrospinal fluid pressure; CSFPp: cardiac-related cerebrospinal fluid pressure pulsations; DCM: Degenerative Cervical Myelopathy; FU: follow-up; HR: heart rate; ISP: intraspinal pressure; MAP: mean arterial pressure; RCT: randomized controlled trial; SCI: Spinal Cord Injury; SCPP: Spinal Cord Perfusion Pressure.

First author, Year, Study Design, Risk of Bias	Region	Population; timing of surgery	Sample size	Intervention (duration [days])	Main outcomes	Surgical approach	Adverse events related to CSFP assessments	Antibiotics	Key findings
Kwon et al., 2009, Single-center RCT	CAN	C-T- SCI AIS A-C (AIS A/B: 83%); within 48 h	24 (2 discarded due to catheter dislocation)	CSFD with target CSFP 10 mmHg (3d)	CSFP pre-to-post decompression, relationship between SCPP and MAP, CSFP	13/22 posterior 6/22 anterior 3/22 combined	1/22 transient headache	Cefazolin or vancomycin postoperatively for 72 h	CSFP increase and waveform restoration post-operatively, CSFD did not reduce CSFP
Squair et al.∗, 2017, Multicenter prospective case-control study	U.S./CAN	C-T-L- SCI AIS A-C (A/B: 82%); within 48 h∗∗	92∗∗	CSFP measurements only (5d)∗∗	Relationship between CSFP, MAP, SCPP and AIS conversion at 6-months FU∗∗	Not reported∗∗	None∗∗	Not reported	SCPP>50 mmHg was a strong predictor of neurological recovery
Altaf et al.∗, 2017, Single-center cross-over interventional study	U.S./CAN	C-T- SCI AIS A-C (AIS A/B: 82%); within 48 h	11	Target MAP 80–85 mmHg through norepinephrine or dopamine (3-5d)	Differential effects of vasopressors on CSFP, MAP and SCPP	Not reported	None	Not reported	Dopamine was associated with higher CSFP than norepinephrine, while MAP remained constant, thus reflecting lower SCPP
Squair et al., 2019∗	U.S./CAN	∗∗	∗∗	∗∗	Relationship of CSFP, MAP, SCPP, and neurological outcomes	∗∗	∗∗	Not reported	SCPP was a better indicator of neurological recovery than MAP
Yue et al., 2020, Single-center prospective interventional study	U.S.	C-T- SCI AIS A-D (AIS A/B: 27%); within 24 h	15	Target SCPP ≥65 mmHg, standard of care (5d)	Safety and feasibility	13/15 posterior 1/15 anterior (1 patient had no surgery)	None	Not reported	SCPP evaluation through CSFP was safe and feasible in a U.S. trauma center
Hogg et al., 2020, Single-center interventional study	U.K.	C-T- SCI AIS A-C (AIS A/B: 69%); within 72 h	13 (in 3 lumbar drain stopped working)	CSFD (10 ml per time, max. 30 ml per day) (NB: catheter insertion at end of surgery) (4-5d)	Effects of CSFD on ISP and CSFP, SCPP (opt.), sPRx computed through CSFP or ISP	9/13 posterior 4/13 combined	4/13 CSF leak (need suturing) 1/13 wound infection 6/13 pseudo-meningocele (6 weeks MRI finding)	Not reported	ISP was pulsatile but CSFP low-pulsatile CSFP with variations over time; CSFD did not reduce CSFP
Zipser et al., 2022, Single-center prospective observational study	CHE	DCM (mild-moderate); mean symptom duration 12 months	19 (2 discarded, 1 to catheter placement, 1 to data quality)	CSFP Measurements only (1d)	CSFPp pre- to post-decompression Relationship of CSFPp and CSFP, MAP, HR, surgical events	6/17 posterior 11/17 anterior	2/17 self-limiting post-puncture syndrome 1/17 toxic skin lesion related to disinfectants	Not reported	CSFPp increased with surgical decompression; Adverse surgical events and confounders could be discerned
Kheram et al., 2022, Prospective case series	CHE	DCM (mild- moderate)	7	CSFP bedside measurements only	CSFP, CSFPp, Queckenstedt's test, relation to MRI	5/7 anterior; 2 no surgery	None	Not reported	One non-operated patient and one patient with residual stenosis had relative CSF block
Kheram et al., 2022, Prospective case series	CHE	C-T- SCI AIS A-D (AIS A/B: 45%); subacute and chronic SCI	11	CSFP bedside measurements only	CSFP, CSFPp, Queckenstedt's test, relation to MRI/CT-myelopgraphy	5/11 posterior 1/11 anterior 3/11 combined; 2 non-traumatic SCI	None	Not reported	CSFPp was reduced in some patients, and indicators of cerebrospinal compliance different compared to a spine-healthy cohort
Kumar et al., 2023, Single- center prospective observational study	IND	T- SCI AIS A-B (AIS A/B: 100%); within 7 days	20	CSFP measurements at lumbar level and above the level of injury (2d)	CSFP above and below level of injury	20/20 posterior	None	Ceftriaxone 1 mg and Amikacin 500 mg 2x/d for 5d	Below level of injury sinusoidal CSFP curve post-decompression, but not before Decrease in CSFP above lesion level, no change in lumbar CSFP
Theodore et al., 2023, Phase 2B multicenter RCT	U.S.	C- SCI AIS A-C (AIS A/B: 64%); within 24 h	11	MAP elevation to 85–90 mmHg±lumbar CSFD with target CSFP <10 mmHg (5d)	Mean CSFP and SCPP during CSFD, AIS changes between BL and 6-month FU	4/11 posterior 3/11 anterior 4/11 combined	None	None	CSFD reduced CSFP and resulted in improved SCPP, preliminary efficacy (better motor scores)

### 3.2 Clinical trials of CSFP in SCI and DCM: comparator and intervention

**Fig. 3:**
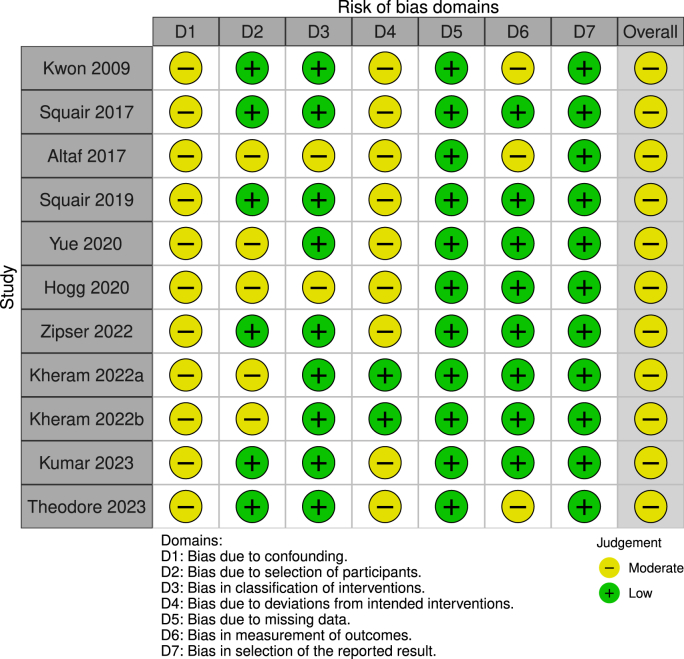
Risk of bias analysis according to ROBINS-I.

The overall risk of bias judgement in terms of safety was considered low to moderate (Fig. 3). Moderate risk was present in most studies with regards to bias due to confounders (ROBINS-I domain- D1), deviations from intended interventions (D4), and measurement of outcomes (D6). There was an overall adverse event (AE) rate of 8% related to the lumbar catheter (n = 15/194), with nine symptomatic AEs, six cases requiring immediate therapy, and no AEs in bedside lumbar puncture. Post-hoc evaluation by severity resulted in two serious AEs (SAE; one case of wound infection in a multimorbid patient requiring antibiotics, one case of toxic skin lesion requiring long-term wound care; 1%), seven mild events (four CSF leakages requiring skin suture – no report of headache and three cases with self-limiting post-dural-puncture headache; 4%), and six cases with events of unclear clinical significance (six cases with asymptomatic pseudo-meningocele; 3%). In two studies, prophylactic antibiotics were administered with catheter insertion, in one study no antibiotics were administered routinely, while no information was available for the other studies.

The perioperative catheter insertion was feasible, and data recording was reliable in most cases, with few reports of inability to insert the catheter, dislocation of the catheter, or problems with data quality (n = 7/194, 4%). None of the studies provided data on anesthesia duration or time from start of catheter insertion to measurements. The selected main study outcomes were commonly CSFP, CSFPp, MAP and SCPP. In addition, clinical outcomes and relatedness with CSFP data were studied in the two multicenter studies using AIS conversion at 6-months follow-up (Theodore et al., 2023; Squair et al., 2017). All studies involving SCI patients investigated SCPP (approximated as the arithmetic difference between MAP and CSFP) and its course during standard of care, or its response to CSF drainage or specific pharmacological intervention. Regarding comparators to CSFP, one intraoperative study co-registered ISP and compared CSFP/ISP to MRI findings (i.e., changes in the longitudinal extent of T2-weighted spinal cord hyperintensities) (Hogg et al., 2020), and two bedside studies provided a qualitative case-based comparison to the degree of spinal canal narrowing and spinal cord tissue signal from MRI/CT-myelography (Kheram et al., 2022, 2023). The studies had the common finding that mean CSFP and/or CSFPp was low at baseline and increased in most cases following surgical decompression (Zipser et al., 2022; Theodore et al., 2023; Kwon et al., 2009; Hogg et al., 2020). Studies focusing on spinal hemodynamics had in common that SCPP provided perfusion information beyond MAP and that higher SCPP (i.e., maintaining SCPP above 50 mmHg) was associated with better outcomes (Altaf et al., 2017; Squair et al., 2017, 2019). Further key findings from the clinical trials are summarized in Table 1.

## 4 Discussion

### 4.1 Summary of main findings

CSFP assessment in SCI and DCM is an emerging research topic reflected by a sizable number of publications in the last decade. Preliminary safety and feasibility results are encouraging for severe cervical and thoracic SCI over 24 h and up to five days after injury. Studies were mostly performed in the perioperative setting and investigated SCPP in SCI with, or without, CSF drainage. The remaining studies focused on monitoring of decompression effects.

### 4.2 Safety and feasibility evaluation

The overall rate of AEs was 8%, similar to that observed in patients without DCM or SCI undergoing lumbar puncture (Bezov et al., 2010; Follett et al., 2000; Aprili et al., 2009). As anticipated, the most common AE was CSF leakage and post-dural-puncture headache (4%), which was generally mild and self-limiting. Nevertheless, SCI and DCM patients enrolled in CSFP monitoring studies need to be fully informed about potential rare SAEs associated with lumbar catheters. From extensive experience of CSF drainage in endovascular aortic repair, where it is used to prevent spinal cord ischemia, there are reports of spinal hematoma and intracranial hemorrhage (Plotkin et al., 2021; Rong et al., 2018). In the studies included in this review, two SAEs were reported. One was a toxic skin lesion due to remnants of the disinfecting agent, which could be prevented through careful inspection of skin and removal of the remnants after catheter insertion. The other was a wound infection in a diabetic patient. While no arachnoiditis and meningitis were reported, these are potential SAEs that patients need to be informed of, in particular when predisposing factors are present (e.g., diabetes) (Hussein et al., 2019). To our knowledge, there is insufficient data to evaluate the benefit of routine antibiotics for preventing catheter-associated infections.

As the exact timing and intervals of safety assessments were rarely reported, some mild events, such as temporary headache, might have occurred undetected. Although less likely, AEs occurring after removal of the catheter might have been underreported. To improve the quality of safety data, the interval and duration of AE assessments should be made available. Of note, there was incomplete reporting on the time of catheter insertion relative to start of anesthesia (i.e., whether insertion was done before or after patient sedation). This information is required to further determine the safety of catheter insertion in sedated patients versus awake patients. The definitive safety profile of intraoperative and bedside CSFP assessment should be evaluated against its diagnostic and therapeutic benefits, which cannot be determined at this stage.

Regarding feasibility, there were few cases (4%) in which catheter insertion was not possible or data quality was insufficient. This is a promising finding, given the expected challenges of catheter insertion in acute and critically ill patients with limited capacity for mobilization. Key indicators of feasibility in the reviewed studies are the execution of multi-center trials, the availability of various measurement systems that allowed for CSFP recording, and the conduction of trials in different parts of the world (i.e., North America, Europe, and Asia). However, most studies were limited to small cohorts (10–20 patients), likely indicating the challenges of performing such interventions. For a more precise estimation of feasibility, more data is needed, including reporting of anesthesia duration (i.e., to what degree catheter insertion lengthens surgery time), screening for pharmacological effects (e.g. muscle relaxation, anesthesia, narcotics), monitoring of potential confounders (e.g., blood CO_2_ levels or pCO_2_, and metabolic changes due to shifts in the pH) (Kheram et al., 2024), and a complete reporting of technical difficulties (i.e., related to catheter insertion and data quality).

### 4.3 Surgical considerations for CSFP monitoring in SCI and DCM

Although the translational development of CSFP monitoring is still in its early stages, it shows potential for several clinical applications. Notably, there is great interest in using CSFP-dependent calculations of SCPP in SCI. Conceptually, and based on preclinical data, there is strong evidence for the value of individually optimizing SCPP to support recovery in SCI and/or prevent intraoperative complications. However, hemodynamic monitoring of the spinal cord is still based on generic MAP targets derived from studies in traumatic brain injury and SCI. Current hemodynamic management guidelines for SCI recommend augmenting MAP to at least 75–80 mmHg but not beyond 90–95 mmHg (Kwon et al., 2024). For a detailed efficacy analysis of interventional trials for optimization of SCPP, which include CSFP-related studies, refer to the updated systemic review of Evaniew et al. (2024), and the systematic scoping review of Gee and Kwon (Gee et al., 2022). Notably, the ongoing Canadian-American Spinal Cord Perfusion Pressure and Biomarker Study (CASPER) further investigates the clinical utility of SCPP maintenance and CSF drainage (NCT03911492).

The second common rationale for CSFP assessments is to help evaluate the extent of surgical decompression. In both DCM and SCI, adequate decompression of the spinal cord is a goal of surgery where nowadays, MRI is the gold standard of assessment despite several limitations. Firstly, MRI is rarely available intraoperatively to inform surgical strategy in real-time. Secondly, early post-traumatic MRI can be difficult to interpret and, particularly for SCI, the extent of spinal compression may evolve as the spinal cord swells. Findings from Kwon (Kwon et al., 2009), Zipser (Zipser et al., 2022), and Kumar (Kumar et al., 2023) demonstrate CSFP as a real-time alternative, with the return of CSFPp suggested to confirm sufficient decompression. A direct comparison between CSFP in DCM and SCI is limited by differences in pathophysiology. This includes, but is not limited to, a higher degree of cord swelling and edema in SCI and more severe damage to the autonomic nervous and vascular systems. In addition, respiratory modulation of CSFP varies between DCM and SCI due to different degrees of respiratory muscle paresis (Berlowitz et al., 2016; Elliott et al., 2024). This is exemplified by findings of Kheram et al., where the increase in lumbar CSFP during the Valsalva maneuver was smaller in adult SCI patients than in spine-healthy controls (Kheram et al., 2023). These suggest that CSFP dynamics might respond differently in SCI and DCM.

Ultrasound is frequently used for intraoperative monitoring of surgical decompression. In SCI, a “free floating spinal cord within the cerebrospinal fluid” should indicate that the spinal cord is free from contact with the anterior elements, suggesting sufficient decompression (Tat et al., 2022). In DCM, inadequate spinal canal expansion following decompression observed using intraoperative ultrasound has been linked to worse neurological recovery (Chen et al., 2021). However, ultrasound cannot be used with all surgical strategies (due to the probe size), is operator dependent, and cannot penetrate bone and therefore does not provide images beyond the margins of the operating field. Both CSFP and ultrasound might be used complimentarily as they provide a functional and a structural perspective. Therefore, studies with parallel ultrasound and CSFP recordings would be of great value. In short, whilst important knowledge gaps remain for CSFP monitoring, it may provide solutions to current, emerging, and potential future needs in clinical care.

### 4.4 ISP and CSFP dynamics

Over the last decade, ISP was advocated by Papadopoulos et al. as a meaningful measure of pressure at the site of injury in SCI (Phang et al., 2015a). Essentially, it is argued that spinal cord swelling in traumatic SCI can lead to separation of the intrathecal space into distinct compartments above, below, and at the level of injury. Following this model, expansion duroplasty was proposed as a potential surgical approach to reduce secondary damage after severe SCI (Saadoun et al., 2016). This is currently being investigated in the randomized controlled multicenter DISCUS trial (Duroplasty for Injured cervical Spinal Cord with Uncontrolled Swelling) (NCT04936620) (Saadoun et al., 2023). Additionally, a North American single-center pilot study is presently recruiting to test the validity of ISP-related measures (NCT04550117) (Dhaliwal et al., 2022). The overall safety and probe placement accuracy was previously confirmed (Phang et al., 2016) and preliminary data were provided for an association between SCPP and neurological outcomes (Saadoun et al., 2017; Chen et al., 2017; Czosnyka et al., 2016; Phang et al., 2015b). Of main interest for this review are the findings from Hogg et al. on the interrelation between CSFP and ISP (Phang et al., 2016). In this study, the pressure transducer was inserted at the level of injury during surgery and a lumbar intrathecal catheter was placed at the end of surgery. This precluded observing the CSFP-ISP interrelation during decompression surgery. CSFP and ISP signals weakly correlated (running correlation coefficient >0.7 for >75% of the time only in 3/13 patients). Theoretically, ISP at any given spinal level and lumbar CSFP are equal in a healthy spine and they only diverge when swelling of the cord results in local compression by the dura. Proof of concept requires more data on simultaneous measurement of both signals in various degrees of cord compression.

### 4.5 Neuroimaging and CSFP dynamics

Three studies investigated the interrelation between structural cervical MRI or CT-myelography and CSFP findings in spinal cord compression (Kheram et al., 2022, 2023; Hogg et al., 2020). In a bedside SCI cohort, spinal cord swelling and spinal canal obstruction as diagnosed from MRI and CT-myelography, respectively, were associated with abnormal CSFP dynamics (Kheram et al., 2023). In the intraoperative SCI cohort of Hogg et al., cord edema was associated with a loss of CSFP-ISP correlation (Hogg et al., 2020). In DCM, by contrast, the degree of spinal canal obstruction from MRI did not correlate with CSFP, hypothetically due to the limited relation between anatomical and pressure readings (Kheram et al., 2022). Although these analyses used small cohorts and a case-based approach, precluding a statement on diagnostic accuracy, the outcomes encourage further research comparing CSFP to neuroimaging. While CSFP, ISP, SCPP, and ICP have potential clinical utility, it is important to note that they require invasive procedures (Evensen et al., 2020; Kim, 2022). At the same time, there is no noninvasive neuroimaging option to directly quantify pressure (Manga et al., 2023). A number of methods that quantify surrogate measures are under investigation (Theodoropoulos et al., 2024; Koshy et al., 2024; Martínez-Palacios et al., 2023; Müller et al., 2023; Dong et al., 2022; Evensen et al., 2018; Zhang et al., 2017; Robba et al., 2016; Padayachy, 2016; Levinsky et al., 2016; Kristiansson et al., 2013; Spiegelberg et al., 2023; Boraschi et al., 2023, 2024; Karimi et al., 2023), but none of them have found entry into routine clinical practice yet (Evensen et al., 2020; Gholampour, 2023) and none of them focus on the spinal compartment.

PC-MRI (Singer, 1978; Bryant et al., 1984; Edelman et al., 1986; Feinberg et al., 1987; Rivera-Rivera et al., 2024), one commonly employed imaging modality (Mehta et al., 2022), can yield time-resolved CSF flow velocities, estimates of CSF pulse wave velocity (Sonnabend et al., 2021; Bessen et al., 2023), measures of stroke volume, and data on spinal cord oscillations. In the spinal canal, a physiologic caudal CSF flow during the cardiac systole, followed by cranial flow during the diastole, has been demonstrated (Enzmann et al., 1991; Beltrán et al., 2024). In DCM patients, a quantitative (i.e., CSF maximum speed) and qualitative (i.e., loss of the bidirectional CSF motion pattern) decrease of CSF dynamics remote from a spinal stenosis at level C1 and C7 was reported (Bae et al., 2017). Lower CSF velocities correlate to the degree of spinal stenosis (Watabe et al., 1999; Vavasour et al., 2014). An alteration of the CSF motion (reflected by a pathologic velocity curve pattern) was associated with a poorer clinical status (i.e., lower Japanese Orthopedics Association (JOA) score) (Bae et al., 2017; Shibuya et al., 2002). Postoperatively, an increase of CSF velocities was demonstrated (Watabe et al., 1999; Tominaga et al., 2002), which correlated with clinical recovery (i.e., only after posterior decompression) (Tominaga et al., 2002). PC-MRI has the potential to add non-invasive information on altered CSF dynamics and increased dynamic mechanical strain to the cord, as in DCM, which may help to identify patients at risk of disease progression, who should undergo decompression.

### 4.6 Preclinical evidence

Although rodent models have been used to study CSF formation, pathway, and absorption sites in healthy conditions (Fehlings et al., 2017; Karimy et al., 2015; Liu et al., 2022) and in the presence of intrathecal occlusion (Berliner et al., 2019; Josephson et al., 2001), their spinal intrathecal space is not suitable for CSFP measurement. Larger animals such as cats, dogs, pigs and sheep, are favoured for spinal CSFP and CSF dynamics investigations. The size of their intrathecal space can accommodate indwelling spinal catheters and pressure transducers <1 mm diameter, and allow sampling of CSF or introduction of sterile fluid, and clinical-grade MRI to measure spinal anatomy and CSF dynamics. Dog, cat and sheep models of cervical epidural intrathecal obstruction have been used to investigate the cranial and spinal compartment distribution of total CSF system compliance (i.e., pressure-volume compensatory reserve) and CSF absorption (Marmarou et al., 1975; Bozanovic-Sosic et al., 2001; Löfgren et al., 1973). In a small number of reports, changes to CSFP and CSFPp, have been observed in supra- and/or infra-stenotic compartments in pre-clinical models of SCI within the sub-acute period (Jones et al., 2012a; Martirosyan et al., 2015; Shapiro et al., 1977).

In a porcine model of traumatic SCI, during 8 h of post-injury extradural compression, supra-stenotic mean CSFP increased while infra-stenotic mean CSFP usually decreased or remained constant, leading to a positive supra-to-infra CSFP differential (Jones et al., 2012a). This compression was presumed to cause complete or near-complete CSF space occlusion. Immediately upon decompression, cranial CSFP decreased while caudal CSFP increased, thus reducing the supra-to-infra CSFP differential. For 6 h following decompression, the supra-to-infra CSFP differential was maintained (Jones et al., 2012a). A companion study observed with ultrasound measurements that spinal cord swelling caused apparently complete (sagittal plane) intrathecal occlusion in eight of 11 animals within this post-decompression epoch (Jones et al., 2012b). In a similar porcine contusion SCI model, without applied residual compression, apparent reduction in lumbar mean CSFP was observed up to 4 h post-injury (Martirosyan et al., 2015). Contusive SCI in cats, with no applied residual compression but presumably producing reduced or occluded intrathecal space due to cord swelling, resulted in increased cisterna magna mean CSFP (+3.7 mmHg) and decreased lumbar mean CSFP (−1.3 mmHg) 4 h post-injury (Shapiro et al., 1977). Together, these observations suggest a dissociation between supra- and infra-stenotic CSFP, associated with loss of communication between the fluid compartments due to intrathecal occlusion at the injury site.

CSFPp was also measured in the aforementioned porcine SCI model (Jones et al., 2012a), and did not change within the supra- or infra-stenotic compartment during extradural compression, or respond consistently to decompression. After decompression, CSFPp increased in the infra-stenotic compartment in 40% of animals, resulting in an increased supra-to-infra stenotic differential (Jones et al., 2012a). In dogs with CSFP measured in the cisterna magna and lumbar subarachnoid space via fluid-filled catheters, a C7 intrathecal (and spinal cord) ligation caused immediate and substantial increase in CSFP and CSFPp in the supra- and infra-stenotic compartments, for 3 h post-ligation (Dunbar et al., 1966). This elevation was interpreted as being due to a sudden reduction in compartment volume on both sides, produced by the ligature. Removing CSF below the ligation resulted in reduced CSFPp, suggesting that reduced pulsation amplitude below an occlusion may result from decreased fluid volume in an isolated spinal compartment (Dunbar et al., 1966).

Phase-contrast MRI (PC-MRI) has been used to non-invasively characterise pulsatile CSF dynamics in pre-clinical models of compressive spinal cord pathologies (Cerda-Gonzalez et al., 2009; Bessen et al., 2025). In a porcine model, decreased speed of bi-directional CSF flow (i.e., peak mean velocity) has been observed cranial and caudal to an induced contusion SCI, with the effect being greatest at the time of peak intrathecal occlusion (Bessen et al., 2025).

The majority of pre-clinical studies have investigated CSFP in the context of traumatic SCI. Since SCI pathology is heterogeneous, it is difficult to elucidate if changes to CSFP in these models are due to only spinal cord compression and swelling, and subsequent occlusion of the subarachnoid space, or are also influenced by potential changes to the mechanical properties of the spinal cord and the surrounding tissues, or disturbed systemic physiology (e.g., cardiovascular complications) often associated with acute traumatic SCI (Bessen et al., 2025; West et al., 2020). Future studies of spinal cord compression without SCI would be beneficial to examine the mechanisms which lead to changes in CSFP and CSFPp. Models of intrathecal constriction/occlusion with and without spinal cord compression have been reported in dogs via epidural balloon catheter (Turk et al., 2006) and lamina-mounted balloons (Löfgren et al., 1973), and in rodent models via a suture ligature (Berliner et al., 2019; Josephson et al., 2001). Adapting these techniques for compatibility with ovine (Trimmel et al., 2022) and porcine (Jones et al., 2012a; Bessen et al., 2025) models currently used for spinal CSF dynamics investigations may yield additional insight.

ISP is assumed to reflect the pressure within the spinal cord and has been used to quantify the magnitude of spinal cord compression. It is measured either between the presumably swollen spinal cord and the dura, or within the parenchyma. ISP has been elevated in a porcine model of traumatic SCI (Streijger et al., 2021), rabbit models of compression SCI via balloon catheters (Yang et al., 2022; Leonard et al., 2015), and in chondrodystrophic dogs with confirmed stenosis (Kunz et al., 2015). Since CSFP or CSF flow dynamics monitoring may provide a method to examine spinal cord compression, a better understanding of the relationship between ISP at the stenosis, and changes to CSFP and CSF flow dynamics in supra- and infra-stenotic compartments, is required. Studies that address such research questions are likely suited to large animal models.

Taken together, these studies demonstrate the feasibility and utility of measuring CSFP and CSF dynamics in pre-clinical large animal models. Although not without limitations, such models offer some advantages over the clinical setting, particularly with respect to the number and complexity of physiological and CSFP monitoring sites possible, and the opportunity for pre-, post-occlusion and longitudinal measures within animals.

## 5 Conclusions

Preclinical findings support the physiologic rationale for CSFP assessments in spinal cord compression. Most clinical trials have focused on intraoperative CSFP assessments in SCI to calculate SCPP, facilitate therapeutic CSF drainage, and monitor the effects of surgical decompression. While preliminary safety and feasibility data are encouraging, a conclusive risk-benefit analysis was not possible, as the clinical utility of CSFP assessments in spinal cord compression has yet to be demonstrated. Future studies should consider coinvestigation of CSFP dynamics and neuroimaging to determine the added value of invasive CSFP diagnostics.

## Funding

This work was supported by Swiss Paraplegia Foundation (Foko_2019_01); Balgrist Foundation; 10.13039/501100001708International Foundation for Research in Paraplegia (P190); Olga Mayenfisch Foundation; and 10.13039/100000001Swiss National Science Foundation (Project Nr. 182683; Project Nr. 10002350). Several authors of this systematic review were also authors in four of the included studies (NK, MF, MH, MS, VK, AC, CZ).

## Declaration of competing interest

The authors declare that they have no known competing financial interests or personal relationships that could have appeared to influence the work reported in this paper.
